# Widal's Test for Typhoid

**Published:** 1898-09-10

**Authors:** 


### WIDAL'S TEST FOR TYPHOID.

By dint of many experiments the exact position which this test is fitted to occupy in the diagnosis of typhoid fever is gradually becoming apparent. The recent researches made by Dr. Scholtz in the Hygienic Institute at Halle University, as analysed in the *Practitioner*, show—(1) That so far as the mere reaction goes it may generally be obtained in the first and always in the second week of an attack of enteric fever; (2) that the serum retains this property for months and years afterwards; and (3) that, apart from the error which this may induce in the event of such a person being subsequently attacked by some other febrile disease, the reaction is not infrequently obtained when the patient not only is not suffering, but when he has not previously suffered from enteric fever.

This might at first sight appear to take away all useful significance from the reaction as a test for typhoid fever, but careful observations of the amount of dilution which the culture will bear before the reaction is lost in the various conditions in which it occurs has shown that typhoid fever can without serious difficulty be differentiated from all other diseases by the observation of very simple precautions. These resolve themselves into adequate and accurate dilution of the culture fluid, and, occasionally, a repetition of the examination after about a week's interval. A dilution of 1 in 50 seems to be the diagnostic limit, the line of demarcation between conclusive and inconclusive observations. In fact, no disease except typhoid fever has been found to react to a dilution of 1 in 50.

The procedure practised by Scholtz, which is very simple, is as follows: The culture fluid having been kept for six or eight hours at a temperature of 37 deg. C. to ensure the active growth of the bacilli, and the diluent having been prepared by sterilising some simple broth (not a culture fluid in which the bacilli have been killed), he makes a preliminary observation on equal parts of the serum and the fluid culture. Should the result be negative, nothing more is needed; but if positive, he proceeds to examine under the microscope the effects of dilutions of 1 in 30 and 1 in 50, and if the 1 in 50 dilution shows agglutination the case is certainly one of recent typhoid. It is to be noted that in a case of doubt, if the case be one of typhoid successive examinations will show a constant rise in the activity of the serum, while any activity which may exist in non-typhoid cases will at any rate remain stationary.

In recording these results it is only right, however, that we should point out that they are the outcome of empirical observations, dealing merely with one particular disease, and have no relation to those larger questions of the relation between the agglutinating and the immunising properties of different serums which have lately been discussed by Dr. Durham and others.

